# Unveiling the Menace of Serratia fonticola: Rising Pathogenic Threat or Bystander?

**DOI:** 10.7759/cureus.56840

**Published:** 2024-03-24

**Authors:** Radha Kunjalwar, Dipika Shaw, Gargi Mudey

**Affiliations:** 1 Microbiology, Jawaharlal Nehru Medical College, Datta Meghe Institute of Higher Education and Research, Wardha, IND

**Keywords:** polymicrobial infection, bacteremia, multidrug resistance, emerging pathogen, serratia fonticola

## Abstract

A rare human pathogen, *Serratia fonticola *(*S. fonticola*)* *has previously been found to cause skin and soft tissue infections post-trauma. The literature contains limited information regarding its management or sensitivity patterns. We aim to share our findings on *S. fonticola* infections in an area with a high rate of antibiotic resistance. To draw attention to this uncommon and rare infection, we share a case series of *S. fonticola*. The antibiogram revealed that *S. fonticola* in all our cases was multidrug resistant. Two of our five cases had a prior history of road traffic accidents and yielded polymicrobial infections along with *S. fonticola*. The other two were revived successfully with proper antibiotic treatment, though one had glucose-6-phosphate deficiency (G6PD) and the last one was a neonate with pulmonary hypertension who grew *S. fonticola* in blood culture.

## Introduction

*Serratia fonticola *(*S. fonticola*) is a relatively uncommon human pathogen, despite being ubiquitous in the natural environment. It was first identified by Gavini et al. in 1979 and belongs to the *Enterobacteriaceae* family [1]. While environmental strains of *S. fonticola* were identified early on, the clinical significance of this bacterium in human infections remained unclear [2]. As a gram-negative bacterium, it is classified as an opportunistic pathogen, and infections caused by *Serratia* species, in general, can pose treatment challenges [3]. The occurrence of human infections associated with *S. fonticola* has been relatively infrequent, leading to speculation about whether the organism is a true pathogen or merely a coincidental finding [4]. A few reported cases of *S. fonticola* associated with human infection were reported in the medical literature. These cases include various infections such as urinary tract infections [3,5], emphysematous pyelonephritis [6], endocarditis [7], skin infections in the diabetic foot [8], biliary tract infection [9], infection complicating parotid malignant tumors [10], cerebellar abscess [11], knee septic arthritis [12], bloodstream infections [4,12], and even an infection following a bear bite [13]. The instances described here demonstrate how *S. fonticola*, which is often thought of as an opportunistic disease, can produce serious and delayed infections that demand several aggressive surgeries and broad-spectrum antibiotic treatment.

## Case presentation

Case 1

A 22-year-old male sought urgent medical attention at the emergency department, reporting a four-day history of fever, chills, headache, and altered mental state. Subsequently, he was admitted to the medicine ward, where a diagnosis of meningoencephalitis was established. Initial assessments unveiled a hemoglobin (Hb) level of 13.2 gm/dL, a white blood cell count (WBC) of 19,800/mm^3^, a platelet count of 2.3/mm^3^, along with slightly deviated sodium (132 mmol/L) and potassium (4.8 mmol/L) levels. Elevated C-reactive protein (CRP) of 99.01 mg/dL and ESR of 12 mm/hr were noted. Notably, neither chest X-ray nor electrocardiogram (ECG) results showed any irregularities. A thorough systemic examination yielded unremarkable cardiovascular, respiratory, and abdominal findings. During the central nervous system assessment, the patient exhibited drowsiness and disorientation. Guided by the clinical presentation, the patient was administered intravenous acyclovir, lorazepam, and ceftriaxone. To identify potential viral infections (hepatitis C V1, hepatitis C V2) and *Mycobacterium*, cerebrospinal fluid (CSF) analysis was undertaken. However, no evidence of mycobacterial and viral infection was detected in the CSF. Microbiological analysis of the CSF sample, on the other hand, indicated the bacterial growth of *S. fonticola*. CSF analysis showed clear fluid along with raised total leucocyte count (TLC) (80 cells/mm^3^), raised protein (76 mg/dL), and raised glucose (68 mg/dL). On the third day of hospitalization, a magnetic resonance imaging (MRI) scan revealed no acute infarction, hemorrhage, or space-occupying mass lesions. A 2D echocardiogram (ECHO) suggested a potential case of infective endocarditis; however, ventricular systolic function remained satisfactory, with a left ventricular ejection fraction of 60% revealed by a transesophageal echocardiogram. With the initiation of appropriate antimicrobial treatment with injection of ceftriaxone and vancomycin, the patient displayed substantial improvements in his clinical parameters. After the 10th day of the hospitalization period, during which he completed the entire antimicrobial course, the patient was discharged, having successfully recovered from his condition.

Case 2

A two-year-old boy who had a known case of glucose-6-phosphate deficiency (G6PD) complained of hematuria and stomach pain and was brought to the emergency room. These symptoms had arisen approximately six days after a suspected ingestion of a naphthalene ball. The child had previously received two units of packed red blood cells (PRBC) transfusion at another hospital due to a low Hb level of 3.9 gm/dL, a platelet count of 3.71/mm^3^, and a lab diagnosis of *S. fonticola* WBC of 29,580/mm^3^. Upon admission to our medical facility, the child's medical assessments revealed a Hb level of 7.5 gm/dL, a WBC count of 11,000/mm^3^, and a platelet count of 1.94/cumm, lactate levels were measured at 2.0, and a raised CRP of 94.62 mg/L; serum urea was 187 mg/dL, serum creatinine was 3.8 mg/dL. Ultrasound of the abdomen and pelvis revealed bilateral raised cortical echotexture suggestive of acute kidney injury. X-ray chest revealed right-sided moderate pleural effusion along with mild left-sided pleural effusion. Despite thorough urine routine microscopy and culture analyses, no specific findings were detected. However, the culture of an endotracheal secretion sample collected on the third day of admission indicated the presence of *S. fonticola*. Due to the low Hb level, another PRBC transfusion was administered-initial treatment involved intravenous ceftriaxone 500 mg. Subsequent adjustments were made to the treatment regimen based on the results of antimicrobial sensitivity tests. The modified treatment plan introduced intravenous cefoperazone/sulbactam and intravenous gentamycin. Additionally, the child underwent hemodialysis three times given acute kidney injury, and a central line was inserted with parental consent. After seven days, the patient's condition improved. He continued to receive intravenous injections of ceftriaxone. Subsequent monitoring of kidney function revealed normal levels of urea, creatinine, sodium, and potassium. The child's clinical state and biochemical parameters improved after implementing appropriate antimicrobial therapy with intravenous ceftriaxone. Following a comprehensive 12-day treatment period, his condition steadily improved, so the child was discharged from the medical facility.

Case 3

A 20-year-old male, previously having a history of renal tubular acidosis (RTA) was admitted to the emergency department after a road traffic accident. He also had a history of a decompressive craniotomy for subdural hematoma evacuation two months prior. After the road accident, he developed a severe injury, featuring a 3x3 cm fracture in the frontotemporal parietal bone, inflicted by a hard blunt object. Following the accident, the patient was initially placed on ventilator support within the intensive care unit. Upon admission, the patient's vital signs were measured at a blood pressure of 110/50 mmHg and a pulse rate of 110 beats per minute. His Glasgow coma scale (GCS) score stood at E1VTM2. Initial investigations revealed a Hb level of 8.8 gm/dL, a WBC count of 9,200/ mm^3^, and a platelet count of 2.19/mm^3^. A plain brain computed tomography (CT) scan revealed multiple fractures of the frontal bone, and occipital bone, along with diffuse cerebral edema. A diagnosis of meningitis was established upon admission, prompting the initiation of intravenous vancomycin, meropenem, and metronidazole. The patient received meticulous monitoring and extensive management in the intensive care unit (ICU) setting. Using the VITEK-2 (bioMérieux, Marcy-l'Étoile, France) automated system, a culture investigation of the patient's endotracheal secretions revealed the presence of *S. fonticola* around day 14 of hospitalization. Because of anemia, the patient was given two units of packed red blood cells on the 20th day. Additionally, a central line was inserted and maintained for a month. An intracranial drain (ICD) was placed concurrently. Meningitis was confirmed by CSF analysis after a lumbar puncture on the 28th day. Despite rigorous antibiotic treatment, the patient's health continued to deteriorate progressively. The blood pressure dropped to 70/40 mmHg on day 30, and the pulse rate dropped to 30 beats per minute. This was accompanied by the patient experiencing cardiac arrest. In response, atropine and epinephrine injections were given along with three cycles of high-quality cardiopulmonary resuscitation (CPR). Unfortunately, the patient's life could not be saved despite intensive efforts to resuscitate him, and he was declared dead on the 30th day.

Case 4

**Figure 1:**
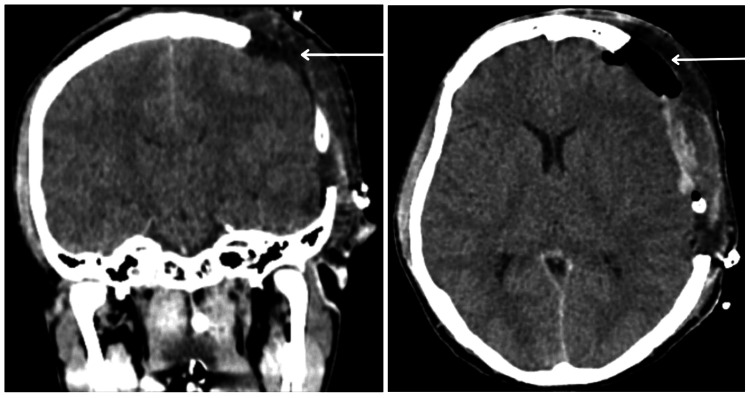
Visible postoperative craniotomy defect in the left temporo-parieto-occipital region

**Figure 2:**
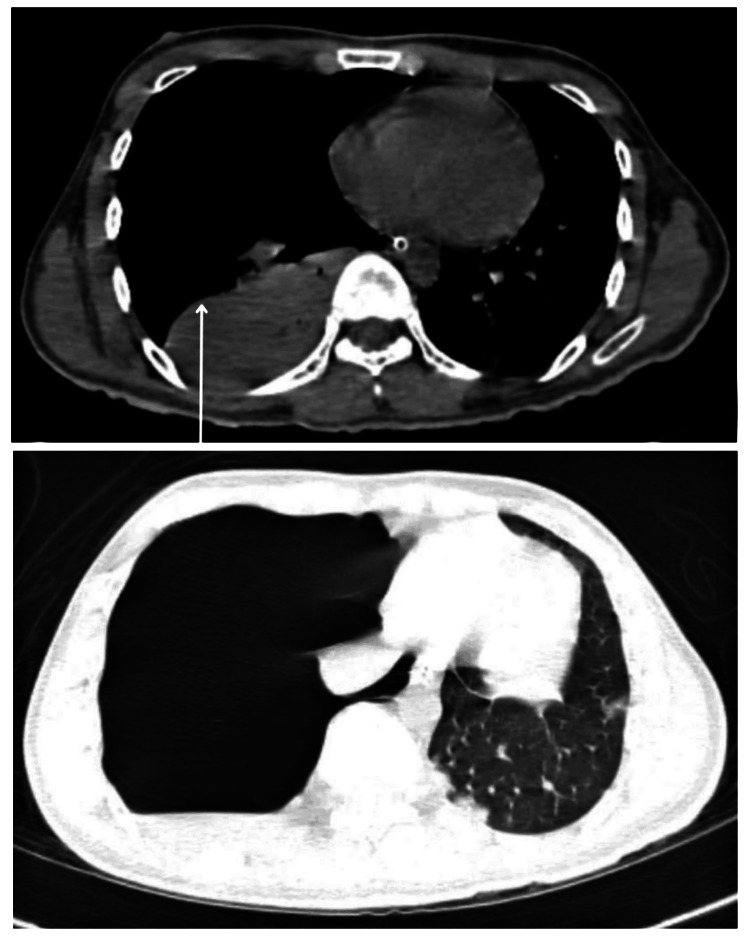
HRCT Thorax revealing right-sided tension pneumothorax along with areas of consolidation remarkably noted superior segment of left lower lobe HRCT- High-resolution computed tomography

A 22-year-old man was brought into the emergency department as a case of a road traffic accident. On the left side of his forehead, a wound measuring 1 cm x 0.5 cm was discovered during the initial inspection. The patient needed to be intubated given his low GCS score of E1V1M2. According to blood tests, Hb levels were 13.6 gm/dL, WBC count was 12,600/mm^3^, and platelet count was 1.12/mm^3^. A subsequent CT scan revealed widespread bilateral cerebral edema, Fax cerebri, and a subdural hemorrhage in the left temporo-parieto-occipital region shown in Figure 1. An uncomplicated decompressive craniotomy was carried out that same day. The patient developed sinus tachycardia and fever following the procedure. High-resolution computed tomography (HRCT) thorax revealed a right-sided tension pneumothorax with the complete collapse of the right lung as shown in Figure 2. On the sixth day, blood culture revealed the presence of *S. fonticola*. The following day, a decline in Hb levels to 7.9 gm/dL and a WBC count reduction to 7,800/mm^3^ was observed. Reintubation was deemed necessary by the eighth day due to a deterioration in the patient's GCS score and respiratory challenges. Subsequent investigation identified hospital-acquired pneumonia, with blood culture outcomes indicating the growth of *Pseudomonas aeruginosa* and *Acinetobacter spp*. On admission, he was given an injection of ceftriaxone; and later an injection of meropenem and vancomycin. Given the prolonged requirement for intubation and the worsening of vital signs, a tracheostomy procedure was conducted. The patient remained in intermittent positive pressure ventilation (IPPV) mode; however, his overall condition continued to deteriorate. Notably, polymicrobial infections emerged over time. Tragically, the patient suffered a cardiac arrest, prompting the initiation of high-quality CPR and administering injections containing atropine and epinephrine. Despite the exhaustive efforts encompassing three cycles of CPR, no palpable pulse or blood pressure readings could be obtained. Despite the valiant attempts to revive the patient, his life could not be sustained, leading to the death.

Case 5

A newborn with respiratory distress was delivered by lower segment cesarean section (LSCS) at 36 weeks to a multigravida mother and admitted to the neonatal intensive care unit (NICU). Despite having an initial Downe score of 2 at birth, breathing difficulties persisted even after oxygen supplementation via nasal prongs. Subsequently, Downe's score increased to 6, reflecting severe respiratory distress, prompting the need for intubation and ventilation. Vital signs registered a heart rate of 144 bpm, respiratory rate of 84/min, and oxygen saturation (SPO2) of 95%. Central cyanosis was evident, while respiratory examination revealed grunting and subcostal retractions; however, other systemic aspects appeared normal. Initial investigations unveiled a Hb level of 13 gm/dL, a total white blood cell count of 12,600/mm^3^, and a platelet count of 1.62/mm^3^. The CRP level was measured at 0.74 mg/dL. A PRC transfusion was administered in response to the low Hb level. Kidney function tests displayed values of 51 mmol/L for urea, 2.1 µmol/L for creatinine, 128 mmol/L for sodium, and 5 mmol/L for potassium. The following day, a 2D ECHO indicated the presence of persistent pulmonary hypertension in newborns. The baby received treatment involving sildenafil, ampicillin, gentamicin, and intravenous fluids. Despite these interventions, weak pulses were observed, and the initiation of epinephrine became necessary. Despite these efforts, the neonate experienced several instances of desaturation. A blood culture was performed on the third day, yielding *S. fonticola*. Alarmingly, this bacterium was multi-drug resistant and sensitive to amikacin, tigecycline, and trimethoprim/sulfamethoxazole as shown in Table 1. The treatment regimen was adjusted to include meropenem, fluconazole, magnesium sulfate, sodium bicarbonate, and vasopressin. Despite these extensive efforts, the baby's condition deteriorated, prompting the transition to a high-frequency oscillatory ventilator (HFO). By the seventh day, repeated blood investigations exhibited a Hb level of 10.4 gm/dL, a total WBC count of 12,400/mm^3^, and a platelet count of 1.98/mm^3^. CRP levels were measured at 4.5 mg/dL, while kidney function tests indicated a urea level of 85 mmol/L and creatinine of 1.9 µmol/L. The neonate received transfusions of PRBC, platelets, and fresh frozen plasma (FFP); however, the condition showed no improvement. Second-line antibiotics, including tigecycline, were administered on the eighth day. During this period, the baby experienced repeated episodes of desaturation, necessitating bag and mask ventilation with 100% oxygen. A sudden episode of bradycardia occurred alongside desaturation, leading to the initiation of CPR with the administration of adrenaline. Despite persistent efforts, the neonate could not be revived, ultimately resulting in the heartbreaking declaration of his passing.

**Table 1:** Antimicrobial susceptibility pattern based on minimum inhibitory concentration (MIC; mg/L) for S. fonticola CSF- Cerebrospinal Fluid ET Secretions- Endotracheal Secretions S- Sensitive, I- Intermediate, R- Resistant

CASES	SAMPLES	PIPERACILLIN+TAZOBACTAM	CEFTAZIDIME	CEFOPERAZONE/SULBACTAMC	CEFEPIME	AZTREONAM	IMIPENEM	MEROPENEM	AMIKACIN	GENTAMYCIN	CIPROFLOXACIN	LEVOFLOXACIN	MINOCYCLINE	COLISTIN	TIGECYCLINE	TRIMETHOPRIM/SULFAMETHOXAZOLE
CASE 1	CSF	64 [R]	8 [R]	32 [I]	≥32 [R]	≥64 [R]	≥16 [R]	≥16 [R]	≥64 [R]	≥16 [R]	≥4 [R]	4 [R]	4 [S]	≥16 [R]	≥8 [R]	≤ 20 [S]
CASE 2	ET SECRETION	≥128 [R]	≥64 [R]	≥64 [R]	≥32 [R]	≥64 [R]	≥16 [R]	≥16 [R]	32 [I]	≥16 [R]	≥4 [R]	≥8 [R]	8 [I]	≤0.5 [S]	≤0.5 [S]	≤ 20 [S]
CASE 3	ET SECRETION	64 [R]	≥64 [R]	≤8 [S]	≥32 [R]	16 [R]	1 [S]	2 [I]	2 [S]	≤1 [S]	≥4 [R]	≥8 [R]	≤0.5 [S]	2 [S]	≤0.5 [S]	≥320 [R]
CASE 4	BLOOD	≥128 [R]	8 [R]	≥64 [R]	≥32 [R]	≥64 [R]	≥16 [R]	≥16 [R]	32 [I]	≥16 [R]	≥4 [R]	≥4 [R]	8 [I]	≤0.5 [S]	≤0.5 [S]	≤20 [S]
CASE 5	BLOOD	≥128 [R]	8 [R]	≥64 [R]	≥32 [R]	≥64 [R]	≥16 [R]	≥16 [R]	4 [S]	≥16 [R]	≥4 [R]	≥8 [R]	4 [S]	≥16 [R]	≤0.5 [S]	≤ 20 [S]

Lab diagnosis of *S. fonticola*

**Figure 3:**
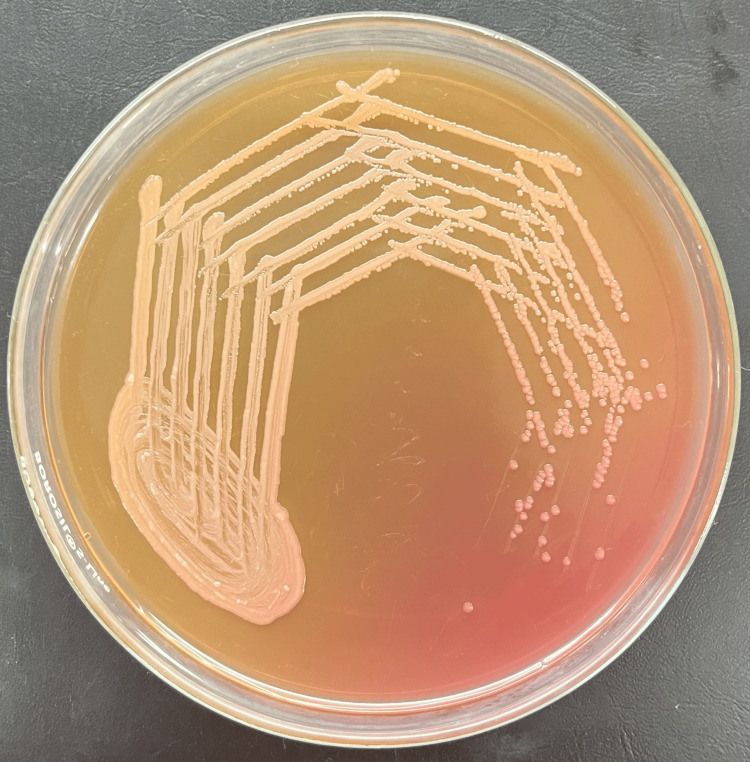
Serratia fonticola species on MacConkey agar Non-lactose-fermenting colonies on MacConkey agar

**Figure 4:**
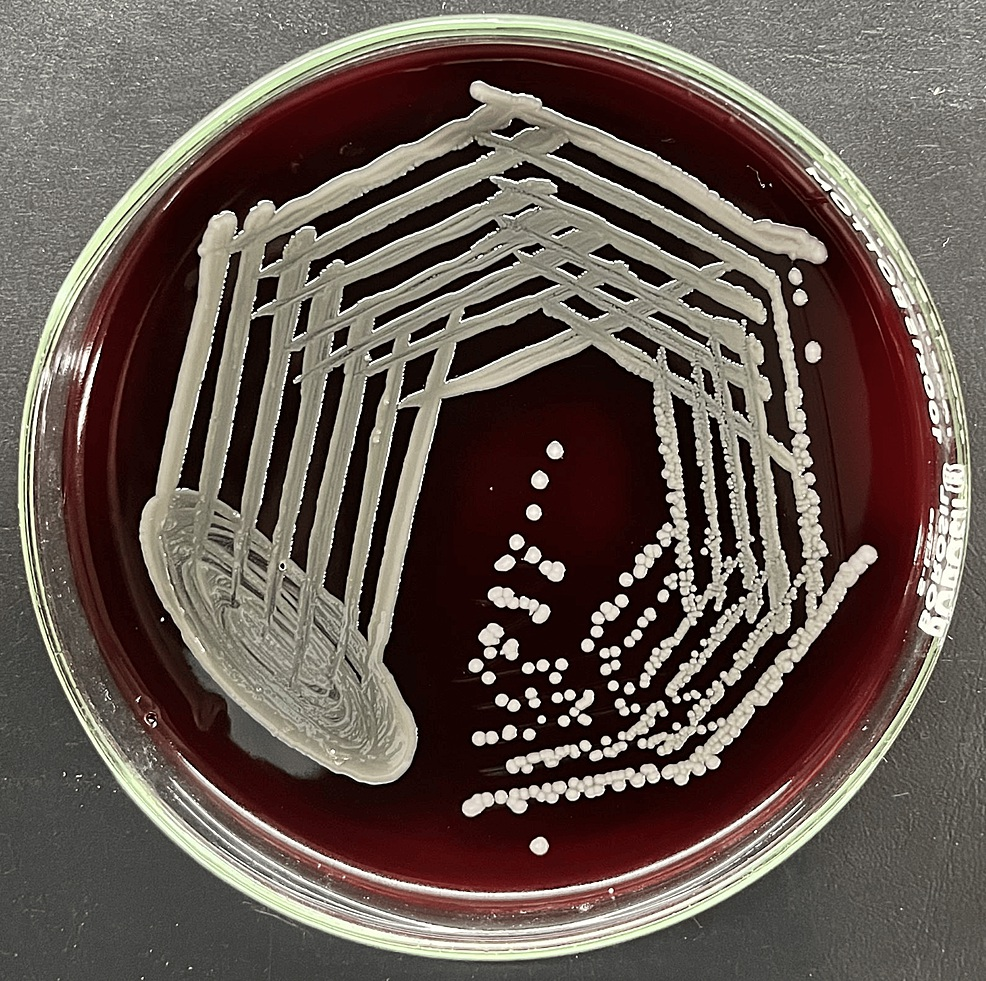
Serratia fonticola species on blood agar Non-hemolytic, greyish-smooth colonies on blood agar

**Figure 5:**
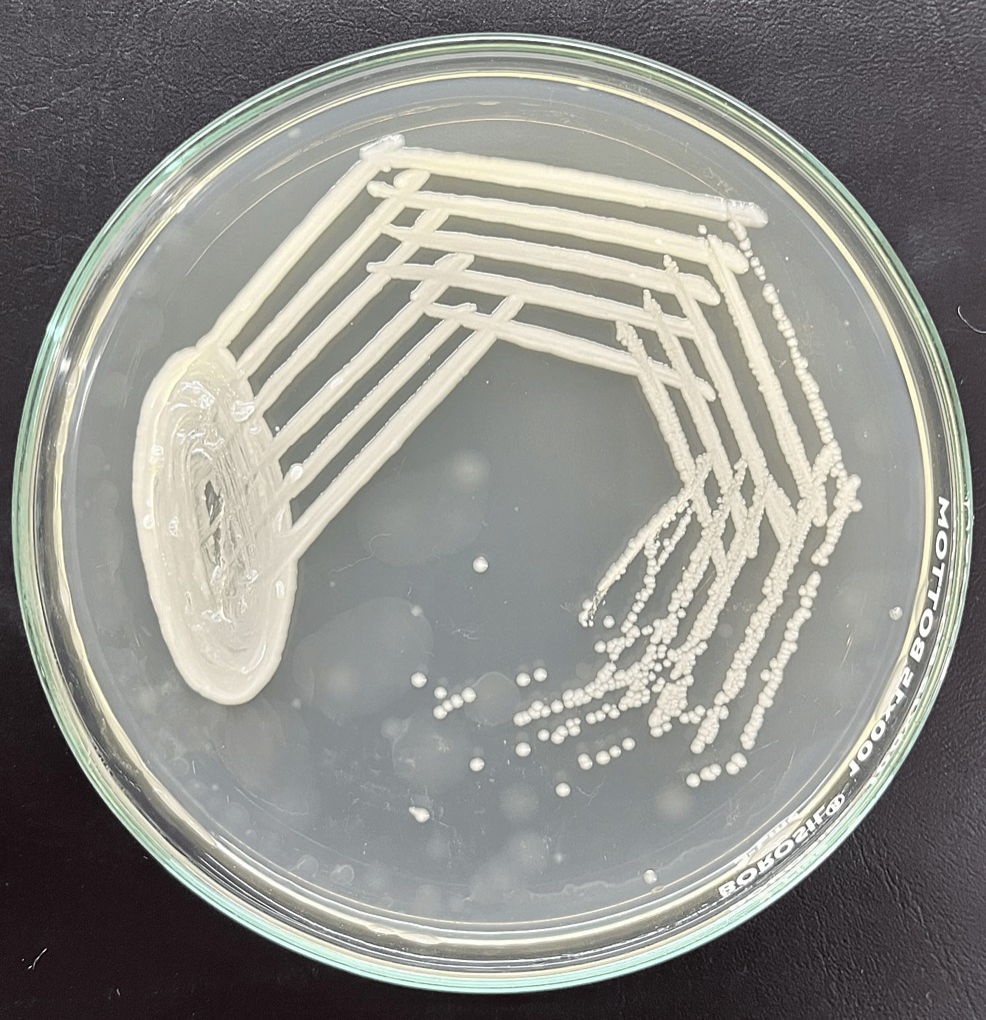
Serratia fonticola species on nutrient agar Opaque-whitish, mucoid, and smooth colonies on nutrient agar

**Figure 6:**
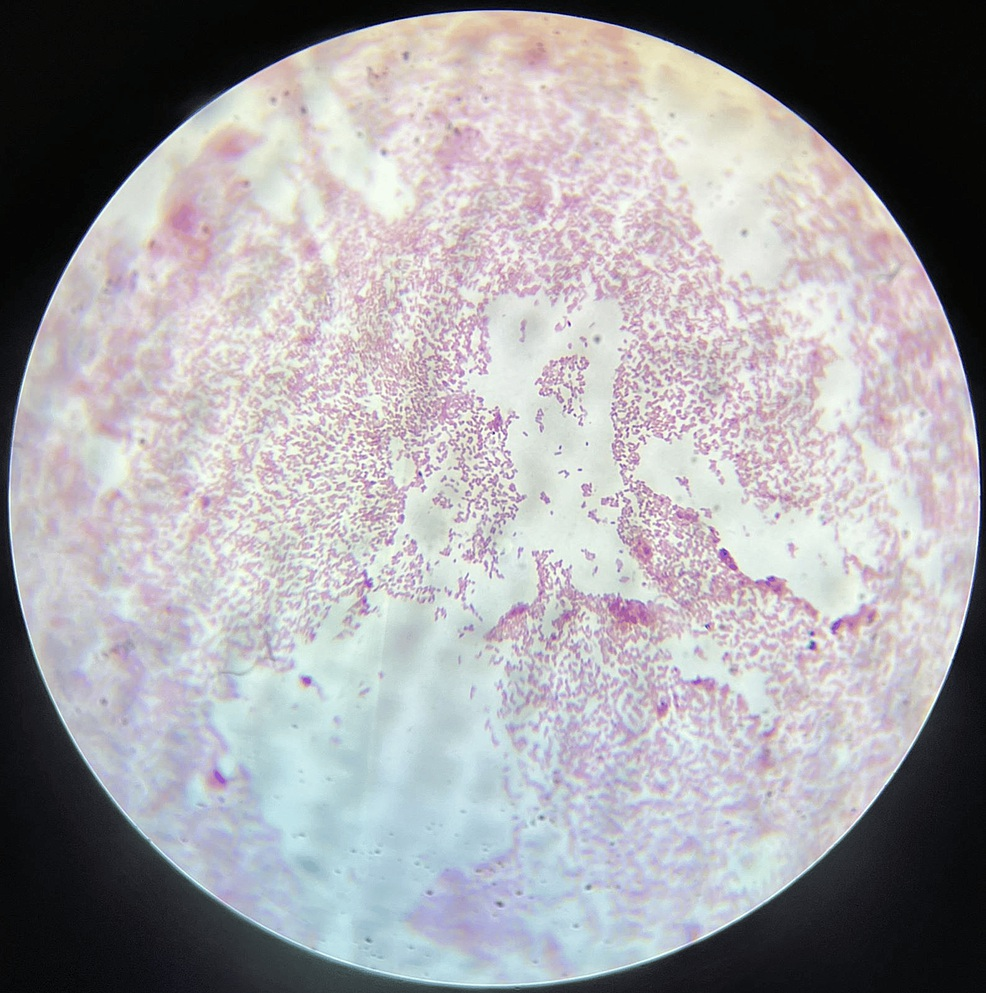
Gram staining revealing gram-negative bacilli

All samples were sent to the Department of Microbiology at the Jawaharlal Nehru Medical College (JNMC), Datta Meghe Institute of Higher Education & Research (DMIHER), Sawangi (Meghe) for microbiological examinations. Gram staining was done. Additionally, the sample was inoculated into MacConkey agar (MA), blood agar (BA), and nutrient agar (NA). Non-lactose-fermenting colonies grew on MacConkey agar, non-hemolytic, greyish-smooth colonies were seen on blood agar and opaque-whitish, mucoid, or transparent smooth colonies on nutrient agar as shown below in Figures 3, 4, 5 respectively. The colony's Gram stain revealed gram-negative bacteria as shown in Figure 6 below. The organism was motile. Based on biochemical reactions shown in Table 2 and colony characteristics, the organism was identified as *Serratia fonticola*. VITEK2 also used an automated technique to confirm identification. VITEK2 is a fully automated system that performs bacterial identification and antibiotic susceptibility testing (AST).

**Table 2:** Biochemical reactions of S. fonticola A/A- Alkaline/Acid reaction H_2_S- Hydrogen Sulphide

BIOCHEMICAL TESTS	RESULTS
Catalase	Positive
Oxidase	Negative
Nitrate	Reduced to nitrite
Hugh Leifson's oxidation-fermentation test	Fermentative
Indole	Negative
Methyl red	Positive
Citrate	Positive
Urea	No hydrolysis
Triple Sugar Iron Test	A/A with gas, no H_2_S
Lysine decarboxylase	Produced
Ornithine decarboxylase	Produced
Arginine dihydrolase	Not produced

## Discussion

Human infections caused by *Serratia fonticola* are rare, and there are minimal documented cases where *S. fonticola* stands alone as the isolated microorganism in cultures from various infections such as bloodstream infections, asymptomatic bacteriuria, diarrhea, skin and soft tissue infections, urinary tract infections, and even brain abscesses. Additionally, polymicrobial groups of organisms are more prevalent, posing a challenge in determining whether *S. fonticola *is the sole cause of infection. Previously, Farmer et al. documented the presence of *S. fonticola* in 11 wound cultures and two respiratory specimens using biochemical analysis [2]. To our current understanding, there have been few reports on isolating *S. fonticola *from respiratory tract samples, and historically, it has been considered an environmental contaminant. Van Hoek et al. detected *S. fonticola *on various commercially available crops, including blanched celery, butterhead lettuce, bunched carrots, iceberg lettuce, spring onions, and radish, suggesting multiple potential exposure routes, including ingestion [14].

Farmer et al. also concluded *S. fonticola* with respiratory tract contaminants [2]. This prompts a debate regarding its role as a pathogen versus a contaminant. However, our recent case study identified this organism in endotracheal secretions in two cases (cases 2 and 3), which raises concerns about its emergence as a potential pathogen. Additionally, it is important to note that in our instances, only two cases (cases 1 and 2) exhibited singular growth of *S. fonticola.* In contrast, the remaining cases showed the growth of *S. fonticola* alongside polymicrobial organisms. This raises a pertinent query regarding whether this organism genuinely serves as the primary cause of infection, emerging as a pathogen, or if it merely acts as a contaminant, serving as a bystander. Addressing this question for future investigations is crucial, warranting newer studies to resolve or draw conclusions on this matter. However, most of the well-known human cases of *S. fonticola* worldwide, are isolated from urine culture [3-5,15]. On the contrary, we did not isolate *S. fonticola *from urinary tract infection (UTI) cases in any of our cases. To the best of our knowledge, in India until now only one case of *S. fonticola* has been isolated from the CSF, and that too from our institute only [16]. Several studies have indicated successful treatment using ciprofloxacin [4,12,17], but our cases demonstrated resistance to ciprofloxacin, levofloxacin, aztreonam, piperacillin/tazobactam, ceftazidime, and cefepime. There was variable sensitivity to cefoperazone/sulbactam, amikacin, colistin, tigecycline, and cotrimoxazole.

Two of our cases saw successful recovery post-treatment with the injection of ceftriaxone (cases 1 and 2). *Serratia spp*. inherently resist antibiotics like ampicillin, amoxicillin, macrolides, nitrofurantoin, colistin, and first-generation cephalosporins, with or without β-lactamase inhibitors. However, these bacteria might develop unique resistance mechanisms against broad-spectrum antibiotics [18]. Timely identification of this infection is crucial due to its inherent resistance to various antibiotics. This is linked to the presence of a FOMA-type and an inducible chromosomal β-lactamase AmpC in *Serratia*. These not only confer antibiotic resistance but also facilitate the transmission of antimicrobial resistance elements to other bacteria, increasing the risk of rapid development into polymicrobial bacteremia or septicemia [6]. Earlier, Gorret et al. reported a patient treated empirically with gentamicin and cloxacillin, later shifting to ciprofloxacin and ceftriaxone after surgery [10]. In our study (cases 2, 3, and 4), invasive procedures were performed, and ceftriaxone was administered. However, only one case (case 2) survived, while cases 3 and 4 resulted in fatalities. Other studies by Villasuso-Alcocer et al. and Mahajan et al. have shown successful outcomes using treatments other than ceftriaxone despite invasive interventions such as surgical removal of a cerebellar abscess and combined endoscopic intrarenal surgery, respectively [6,11].

## Conclusions

In recent years, public health has become increasingly concerned with the growing issue of antibiotic resistance.* Serratia fonticola *may still be a relatively uncommon human pathogen, particularly when it is linked to underlying diseases, invasive, instrumented interventions, or asymptomatic infections, as it was in two of our scenarios. However, its incidence could occur in severe cases in such instances. Also, we discovered *S. fonticola* from two of the endotracheal secretion samples; out of them one had a fatal outcome. Also, two of our patients had road traffic accidents along with polymicrobial infections with fatal outcomes. *S. fonticola* may act as a human pathogen when isolated, which is an infrequent occurrence. This clinical series of cases from our healthcare system suggests that *S. fonticola *might be an emerging unusual human pathogen when isolated alone and might serve as a bystander when found along with more virulent organisms such as in polymicrobial infections. Therefore, more research studies should be done on the same to evaluate the correct identification, and proper sensitivity patterns, to improve the prognosis and positive outcomes along with molecular detection.
